# Supplementary material for: In primary airway epithelial cells, the unjamming transition is distinct from the epithelial-to-mesenchymal transition
Source: Nat Commun. 2020 Oct 7;11:5053. doi: 10.1038/s41467-020-18841-7 (PMC7542457; doi:10.1038/s41467-020-18841-7)
Supplement: Supplementary file 1 — Supplementary Information [file 41467_2020_18841_MOESM1_ESM.pdf]

Supplementary Information

Supplementary Figures

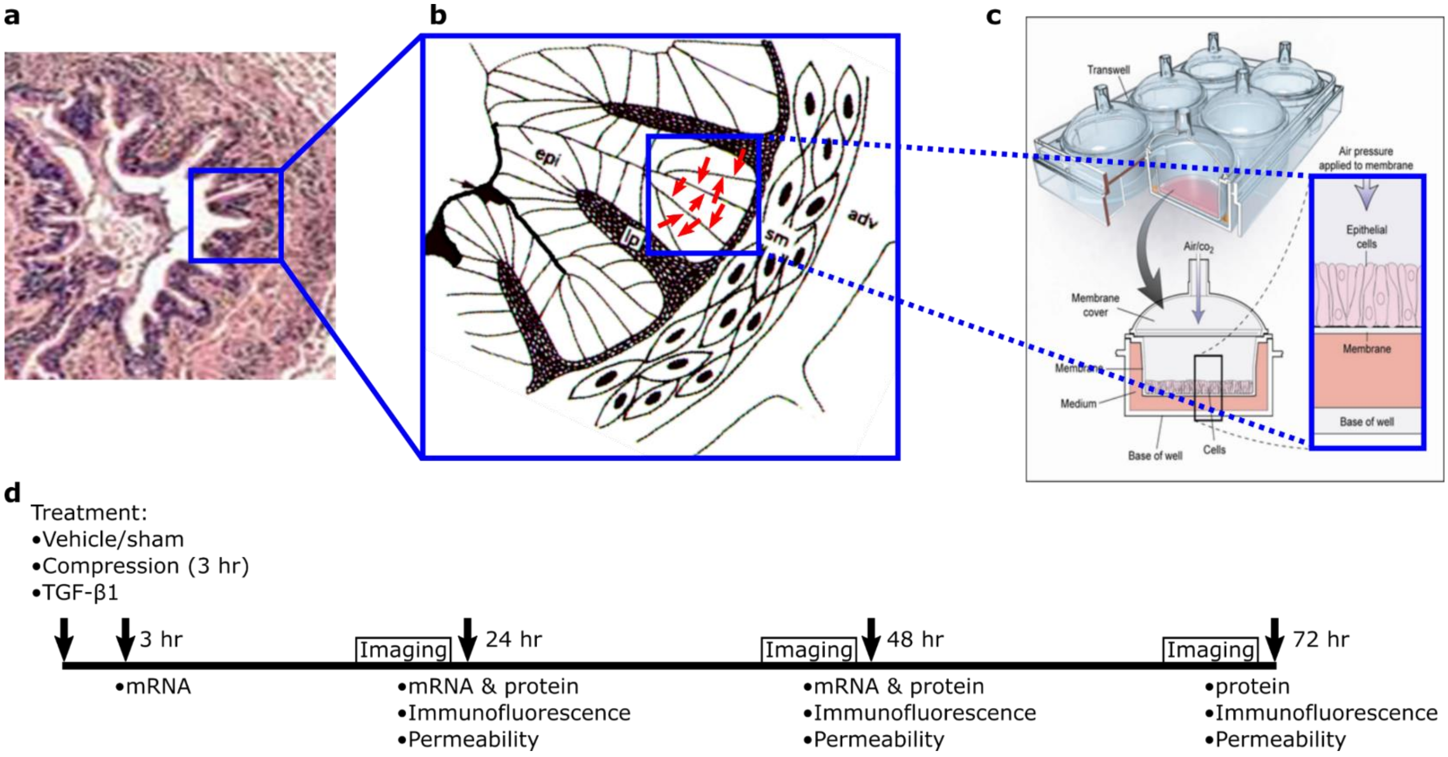

Supplementary Figure 1: Experimental set-up comparing jammed epithelial layer to layers undergoing partial EMT or unjamming.

a. During an asthma exacerbation, the airway narrows, causing the airway epithelium to buckle[1-4]. **b**. The buckled epithelium is mechanically compressed (red arrows), and thus promotes airway remodeling[1, 3, 5-8]. **c**. This mechanical compression of the airway epithelium is stimulated *in vitro* using well-differentiated primary human bronchial epithelial cells. We used 30 cm H_2O because this compressive dose has been previously established to trigger pathologic airway remodeling that is a hallmark of asthma. In our original paper on UJT in airway epithelium[9] we performed dose-response experiments with 0, 10, 20 and 30 cm H_2O, and found a sharp difference in response between 20 and 30 cm H_2O, but no further increase in response between 30 and 40 cm H_2O. This indicates that the sweet spot in the dose-response curve lies between 20 and 30 cm H_2O, with the latter being sufficient to elicit the maximal response. Using this in vitro system, work by our group and others has established that mechanical compression induces events that occur in the remodeled asthmatic airway. These events include increased matrix deposition, goblet cell hyperplasia, and airway smooth muscle hyperplasia and hypercontraction, as well as production of asthma-associated mediators including YKL-40 and tissue factor-containing exosomes[2, 5, 8, 10-16]. All of these pathophysiological events are triggered by mechanical compression in the absence of any inflammatory cells. Our *in vitro* work was validated in humans by Grainge et al[3]. Together, this body of literature has established *in vitro* and in mild asthmatic patients *in vivo* that even in the absence of inflammatory cells, the mechanical effects of bronchoconstriction are sufficient to drive aberrant airway remodeling that is a cardinal feature of asthma. **d**. Time-course outline of the experiments. Well-differentiated primary human bronchial epithelial cells in air-liquid interface culture were exposed to either a vehicle/sham condition, to mechanical compression mimicking the physical forces associated with bronchospasm (a-c), or to TGF-β1 at time t=0 hr. At 3, 24, 48, or 72 hrs following exposure, cells were collected for the indicated endpoint assay, and evaluated for epithelial and mesenchymal characteristics. Time-lapse microscopy images were taken for 2 hours prior to 24, 48 and 72 hr timepoints. **a** – **c** Originally published in Park et al, Nature Materials 2015. Permission requested from Nature Publishing Group.

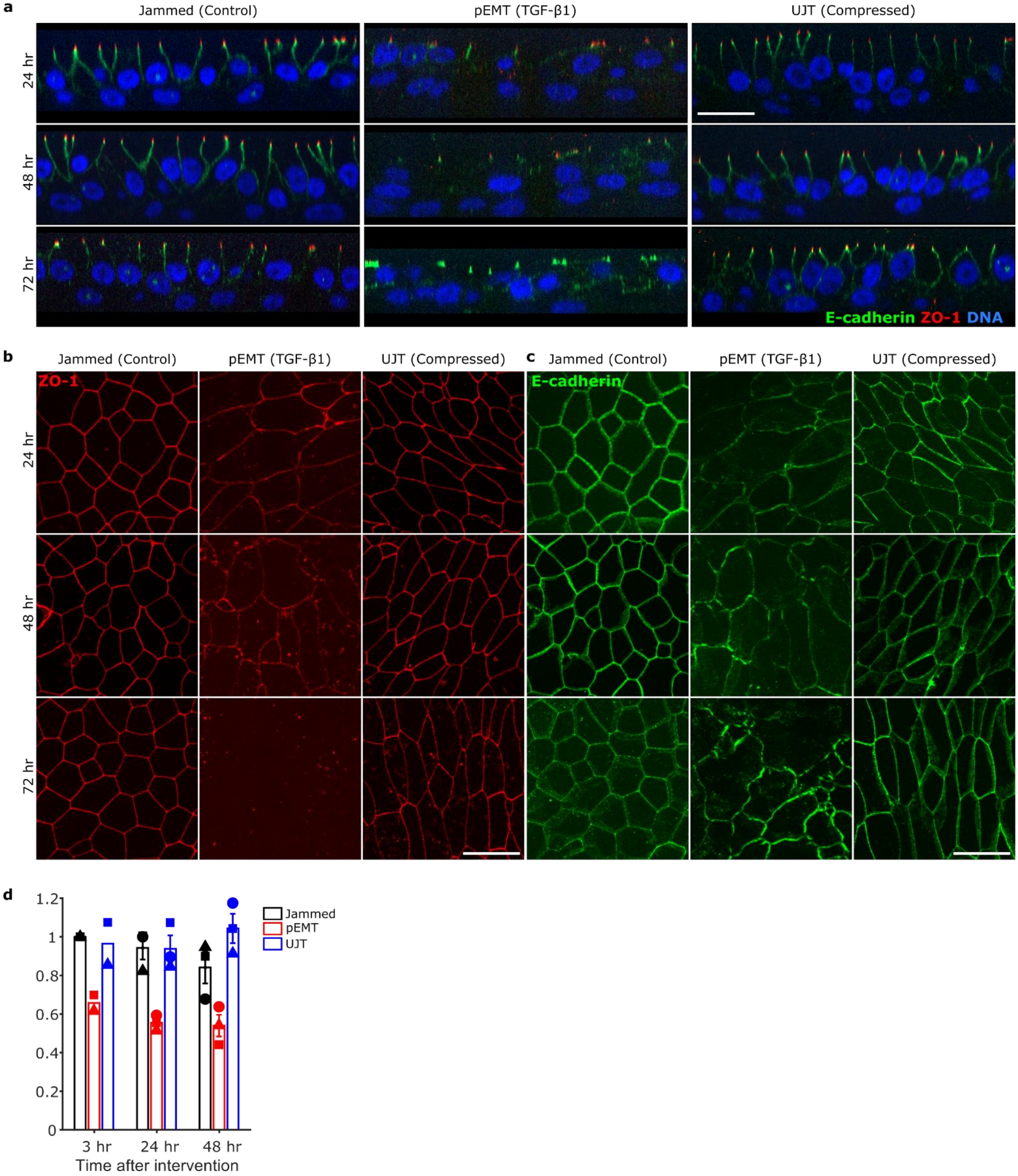

Supplementary Figure 2: Partial EMT reduces epithelial character, while unjamming maintains epithelial character.

Extended data from Figure 2. Representative immunofluorescence (IF) images (**a-c**) at 24, 48 or 72 hrs after stimulus for jammed (control), pEMT (TGF-β1-treated), and UJT (compressed) layers. **a**, At all time points, in both jammed and unjammed layers, ZO-1 (red) is localized at the apical tight junctions, while E-cadherin

(green) is localized at lateral adherens junctions, consistent with the epithelial phenotype; DNA is shown in blue. In pEMT layers, both ZO-1 and E-cadherin are delocalized from apical and lateral junctions, consistent with mesenchymal phenotype. This occurs as early as 24 hrs and persists during pEMT. **b**, **c**, At all time points, in both jammed and unjammed layers, apical tight junctions (ZO-1, **b**) and lateral adherens junctions (E-cadherin, **c**) remain intact, while in pEMT layers the cell edges comprised of these junctions become progressively disrupted. Panels in **a-c** represent an expanded version of data shown in Fig. 2 in the main text; scale bar is 20 μm. **d**, Quantification (n=3 donors) of protein expression of E-cadherin; representative blot shown in Fig 2h. Expression of E-cadherin decreases during pEMT but remains unchanged during UJT. Quantification was made across multiple blots run in parallel.

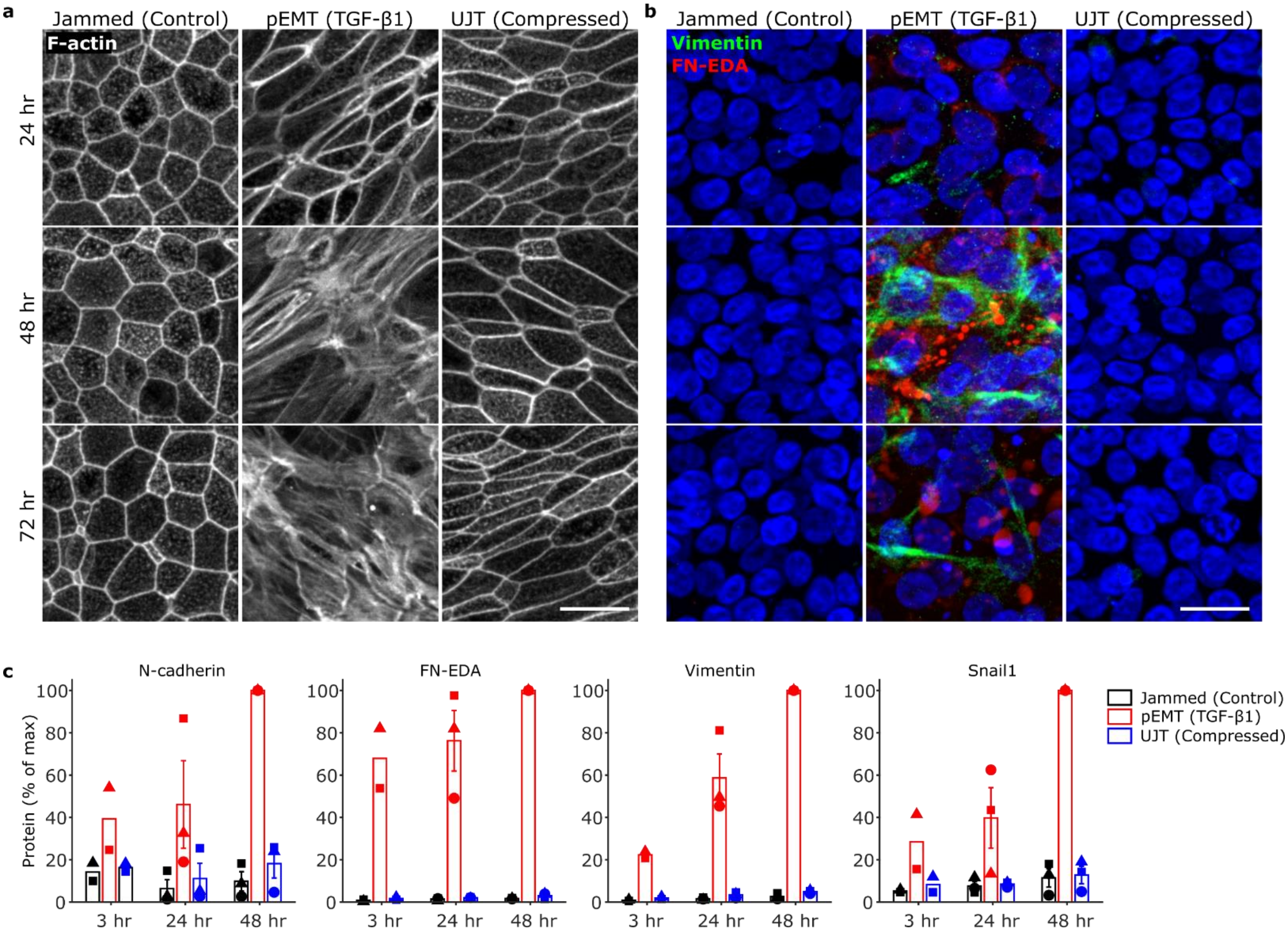

Supplementary Figure 3: Partial EMT increases mesenchymal character, while unjamming does not.

Extended data from Figure 2. **a**, During pEMT, cortical actin becomes disrupted while apical stress fibers emerge, indicating loss of epithelial character and gain of mesenchymal character (at 48 and 72 hrs). During UJT, cells maintain intact cortical F-actin; aside from elongated cell shape, cortical actin in jammed versus UJT was indistinguishable. **b**, IF images stained for mesenchymal makers: cellular fibronectin (the Extra Domain A splice variant, denoted FN-EDA, red) and vimentin (green). FN-EDA and vimentin are expressed during pEMT but not during UJT. Vimentin appears as basally located fibers, while FN-EDA appears as cytoplasmic globules. Panels in **a**, **b** represent an expanded version of data shown in Fig. 2 in the main text; scale bar is 20 μm. **c,** Quantification (n=3 donors) of protein expression of N-cadherin, FN-EDA, vimentin and Snail1; representative blots shown in Fig 2h. During pEMT, mesenchymal markers, N-cadherin, FN-EDA, and vimentin, and the EMT-inducing transcription factor (TF) Snail1, progressively increased. During UJT, these protein levels remained unchanged. Quantification was made across multiple blots run in parallel.

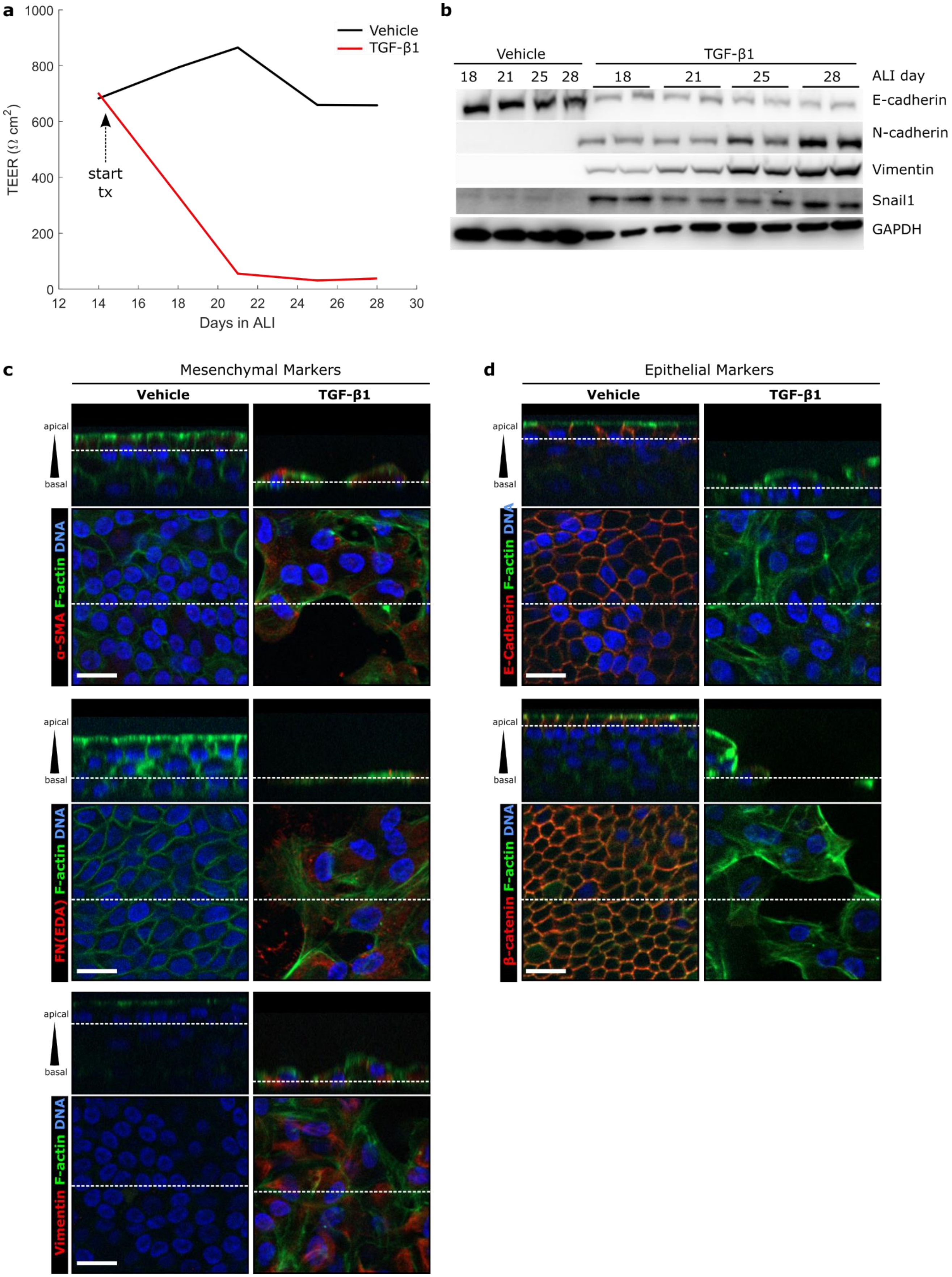

a
1000
800
600
400
200
0
TEER (Ω cm2)
start tx
12 14 16 18 20 22 24 26 28 30
Days in ALI
Vehicle
TGF-β1
b
Vehicle
TGF-β1
18 21 25 28
18 21 25 28
ALI day
E-cadherin
N-cadherin
Vimentin
Snail1
GAPDH
c
Mesenchymal Markers
Vehicle
TGF-β1
apical
basal
α-SMA F-actin DNA
FN(EDA) F-actin DNA
Vimentin F-actin DNA
d
Epithelial Markers
Vehicle
TGF-β1
E-Cadherin F-actin DNA
β-catenin F-actin DNA

Supplementary Figure 4: Long exposure to TGF-β1 is required for HBE cells to undergo full EMT

The main text focuses on the initial events during early partial EMT. To elicit a more complete EMT, involving a complete loss of epithelial character and a strong gain of mesenchymal character and cell individualization, we treated differentiated HBE cells with TGF-β1 (10 ng/ml) continuously for up to 14 days, starting on ALI day 14. Data shown are from a single donor. **a**. Epithelial layer integrity was measured by transepithelial electrical resistance (TEER) every four days starting just prior to treatment with TGF-β1 or a vehicle control. By 4 days of treatment, the TEER of TGF-β1-treated HBE cells undergoing EMT was substantially lowered compared to the control cells. By 8 days of treatment, the TEER of the treated cells was negligible, due to large areas of denuded cells and significant breakdown to epithelial junctions. Shown: average TEER across 2 biological replicates for a representative donor. **b**. To determine extent of EMT over the 14-day treatment, epithelial and mesenchymal markers were detected by western blot in HBE cells treated with TGF-β1 or vehicle. Vehicle-treated HBE cells retained expression of E-cadherin and did not acquire expression of N-cadherin, vimentin, or Snail1. By contrast, TGF-β1—treated HBE cells lost expression of E-cadherin and gained expression of N-cadherin, vimentin, and Snail1. Expression of N-cadherin and vimentin progressively increased over time. **c**. IF images stained for mesenchymal markers (α-SMA, FN-EDA, vimentin) show that expression of these were increased in response to TGF-β1 treatment (14 days), but that they were not expressed in vehicle-treated control HBE cells. **d**. IF images stained for epithelial markers (E-cadherin and β-catenin) shows that expression of these were maintained and localized at the cell-cell junctions in vehicle-treated control HBE cells. In contrast, these epithelial markers were undetectable following TGF-β1 treatment (14 days), where cell contacts were completely disrupted. Scale bars in **c**, **d** are 20 μm.

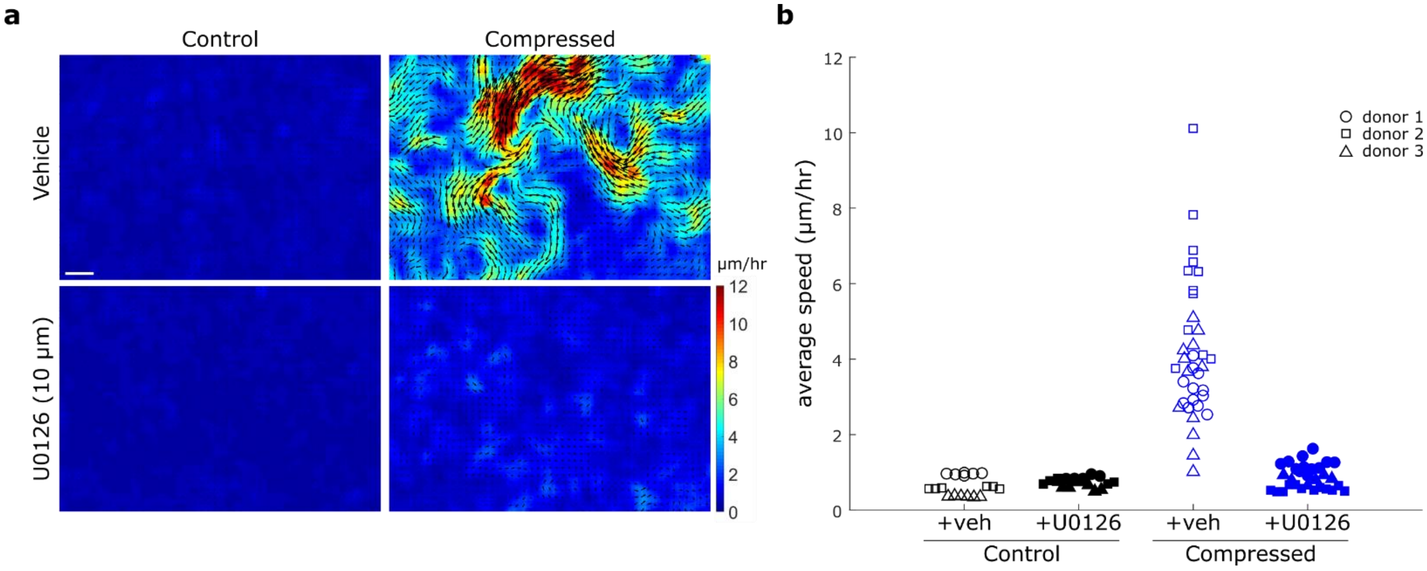

Supplementary Figure 5: ERK signaling is required for compression-induced UJT

Representative speed maps (**a**) and average speed (**b**) for control and compressed HBE layers at 24 hrs, in the presence of vehicle or an ERK inhibitor U0126 (10μM). Average speed was calculated for 6-12 fields of view per condition from n=3 donors. Scale bar in **a** is 100 μm.

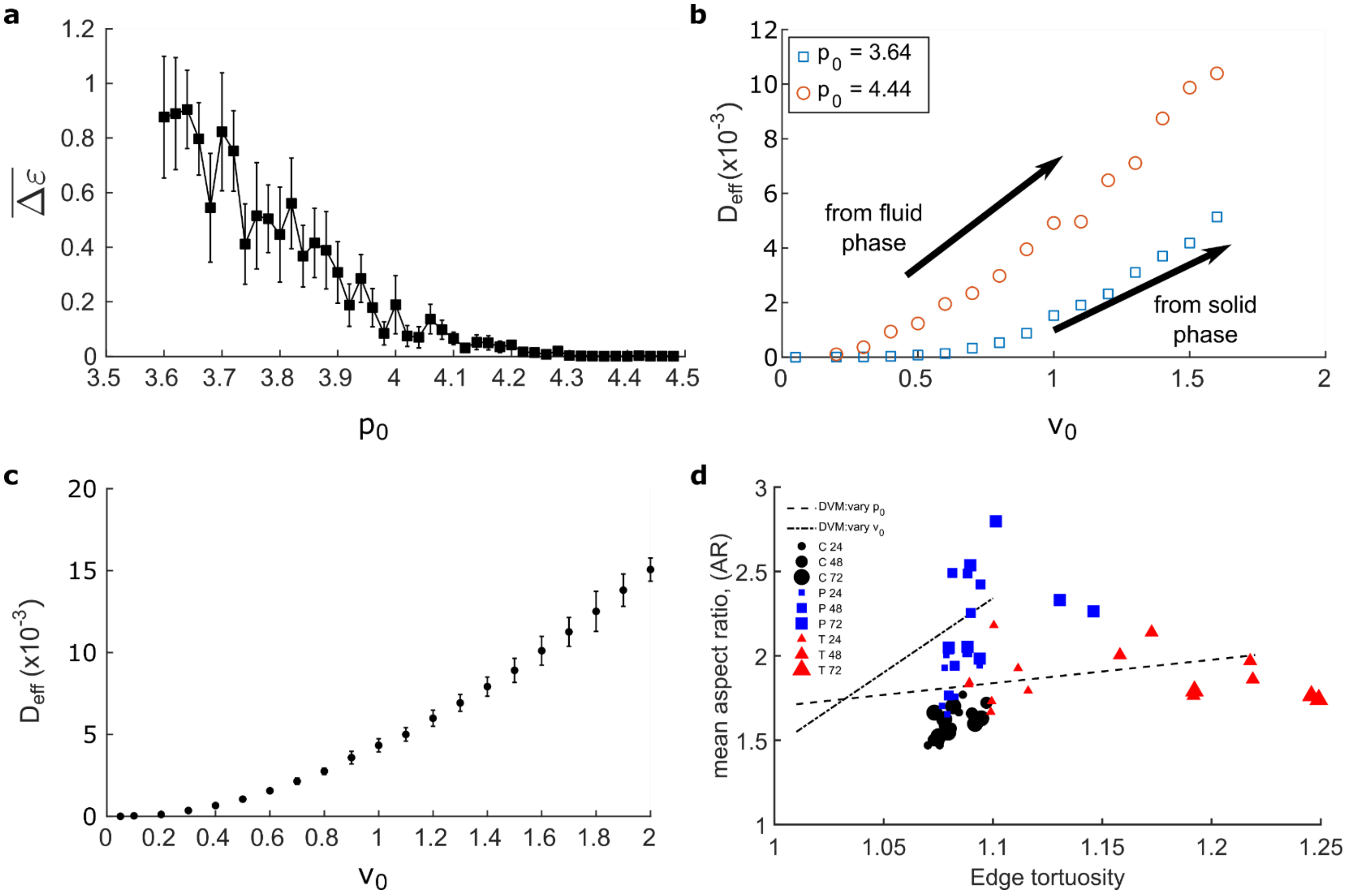

Supplementary Figure 6: Energy barriers and migration in the dynamic vertex model (DVM). a, Average energy barrier to structural rearrangements ($\overline{\Delta\varepsilon}$) for cells in the DVM when p_0 is increased and v_0 is small (v_0=0.05) and cell edges are allowed to curve. Data shown are mean ± SEM for n=10 independent simulations, with 50 T1 rearrangements analyzed in each simulation. Precisely where the edge tensions vanished, near p_0~4.1, the energy barrier drops to zero. This indicates that cells in this fluid-like phase require only a small increase in v_0 in order to become motile, as shown in **b**. This mode of migration appears to correspond to pEMT (see Fig 4g). **b,** The effective diffusivity, D_{eff} (defined as $D_{eff} = \lim_{t\to\infty} \langle \Delta r(t)^2 \rangle / (4t)$) captures the amount of average movement of cells in the DVM and is shown for two representative values of p_0, as v_0 is increased. For cells in in the solid-like phase (p_0=3.64), cellular movement occurs at a substantially higher value of v_0, compared to cells in the fluid-like phase (p_0=4.44), which exhibit vanished tension and vanished energy barriers, as in **a**. **c**, When v_0 is increased and p_0 is moderate (p_0=4.0), cells migrated as shown by increasing D_{eff} (mean ± SD for n=8-20 independent simulations). In this case, cell edges remained under high levels of tension. Though energy barriers to cellular rearrangement were finite at this p_0 (as shown in **a**), cellular migration occurred when cellular propulsion was sufficient to overcome these barriers. This mode of migration appears to correspond to the UJT (see Fig 4g). **d**. DVM predicts that during UJT versus pEMT two different metrics of cell shape diverge; aspect ratio (AR) emphasizes elongation whereas shape parameter q emphasizes perimeter (see Fig 4g). Because perimeter can be increased both by elongation and by edge tortuosity, here we compare model versus experiment for AR and edge tortuosity. Increasing p_0 while allowing edges to curve moderately increases AR but substantially increases edge tortuosity (- - -), resulting in somewhat elongated cells with tortuous edges. By contrast, increasing v_0 (-·-·-) substantially increases AR but minimally increases edge tortuosity, resulting in elongated cells with straight edges. Measurements of cells

undergoing pEMT (▲) are consistent with the predictions for increasing p_0. However, measurements of cells undergoing UJT (■) or in the jammed state do not line up as strongly with predictions from the model, due to the existence of a baseline curvature in cell edges in experiments that is not captured in the model.

Supplementary Tables

Supplementary Table 1. Biophysical measurements show how pEMT and UJT diverge.

Condition	Timepoint (hrs after treatment)	Average speed (μm/hr)	D_{eff} (μm^2/hr)	Mean aspect ratio (AR)	Structural pack size (#cells)	Dynamic pack size (effective diameter, μm)
Jammed (Control)	24	1.0 (0.4)	1.6 (1.3)	1.58 (0.07)	5 (2.3)	38 (17)
	48	2.8 (1.2)	8.3 (3.5)	1.59 (0.06)	4 (0.3)	107 (40)
	72	2.1 (1.0)	7.9 (4.1)	1.60 (0.04)	3 (0.7)	83 (36)
pEMT (TGF-β1)	24	3.0 (0.8)	6.3 (2.5)	1.84 (0.08)*	10 (2.3)	223 (67)
	48	2.4 (0.4)	5.0 (1.3)	1.92 (0.09)*	4 (1.8)	125 (62)
	72	1.9 (0.3)	3.6 (1.1)	1.83 (0.06)	2 (0.5)	35 (4)
UJT (Compressed)	24	3.7 (0.9)*	9.0 (3.0)	1.82 (0.08)*	45 (22)	115 (36)
	48	9.9 (2.0)*#	64.2 (19.7)*#	2.22 (0.12)*	110 (38)*#	339 (54)*
	72	9.5 (3.0)*#	97.8 (37.6)#	2.30 (0.09)*#	237 (45) *#	328 (74)*#

Dynamic and structural metrics for HBE cells undergoing pEMT or UJT reported as mean across donors with standard error in parentheses. For dynamic measurements (speed, D_{eff}, dynamic pack size), data was obtained from n=4 donors per time point and condition captured in independent experiments. For structural measurements (AR, structural pack size), data was obtained from n=3 donors per time point and condition captured in independent experiments. Statistics are shown as follows: *p<0.05 vs. control; #p<0.05 UJT vs. pEMT. Statistical significance was determined by a one-way ANOVA followed by post-hoc multiple comparisons tests with Bonferroni correction.

Supplementary Table 2. Primers used in qPCR to determine mRNA expression of mesenchymal markers.

GAPDH	FW	5'-TGGGCTACACTGAGCACCAG-3'
GAPDH	RV	5'-GGGTGTCGCTGTTGAAGTCA-3'
FN(EDA)	FW	5'-GAGCTATTCCCTGCACCTGATG-3'
FN(EDA)	RV	5'-CGTGCAAGGCAACCACACT-3'
VIM	FW	5'-TGTCCAAATCGATGTGGATGTTTC-3'
VIM	RV	5'-TTCTACCATTCTTCTGCCTCCTG-3'
ZEB1	FW	5'-GATGATGAATGCGAGTCAGATGC-3'
ZEB1	RV	5'-ACAGCAGTGTCTTGTTGTTGT-3'

Supplementary Methods:
Simulation of collective cellular behavior using an extended Dynamic Vertex Model

Vertex-based models[17-19] have been successful in describing cell shape statistics in developing embryos[20-23] and glassy and jamming behavior in cultured epithelial sheets[9, 24-26]. These computational models are simple yet contain the salient features that offer useful means to investigate the role of cell-cell interactions and how single cell behavior is connected to emergent collective behavior at the multicellular level.

The framework of this model is based on the observation that the cells comprising epithelial sheet tend to maintain a columnar structure and the 2D cross-section of the epithelial sheet forms a polygonal tiling in the plane[17, 18]. The apical surface of cells is then specified by the location of vertices, as well as the geometry of edges that connect the vertices. In this model, the edges represent the conformation of the cell-cell junctions. Unlike classical implementation of the vertex model, here we allow the edges to be curved as an emergent property (as detailed below). The cells in such a tiling emerge as *curved* polygons of different shapes with well-defined area (A) and perimeter (P).

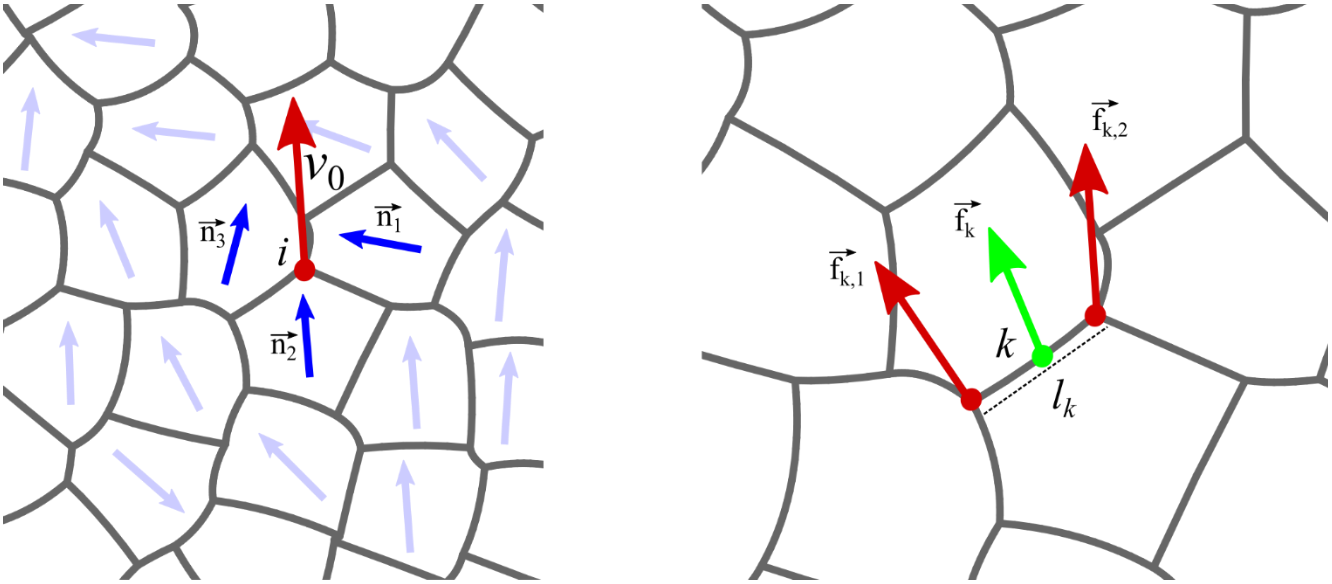

Figure S1. Schematic for the dynamic vertex model which shows a part of the simulated tissue. Left, the net propulsion (solid red arrow, definition Eq. 3) on a given vertex (red dot) has a magnitude v_0 and direction derived by the polarizations of three cells sharing the vertex, given by $\vec{n_1}$, $\vec{n_2}$ and $\vec{n_3}$ (solid blue arrows). Right, the active force on a mid-edge vertex k given by $\vec{f_k}$ (solid green arrow, definition Eq. 6) which depends on the active forces on the head vertex ($\overrightarrow{f_{k,1}}$) and tail vertex ($\overrightarrow{f_{k,2}}$) (solid red arrows), l_k being the end-to-end length of the edge carrying k.

<u>The classical vertex model:</u> Beginning with the network of vertices and connecting edges, vertex models then describe the mechanical energy of each cell written as function of cell area (A_i) and perimeter (P_i) of the i-th cell:

$$E_i = K_P {P_i}^2 + \lambda_i P_i + K_A \left(A_i - A_0^i\right)^2 \qquad (1)$$

The first term describes the elastic energy required to deform the cell cortex and the contractile elements embedded within the cell cortex. This term is quadratic in cell-perimeter, $K_P {P_i}^2$ with elastic constant K_P. The second term describes the energetic effects arising from cell-cell adhesion and cortical contractile tension. This term is linear in cell perimeter, $\lambda_i P_i$. The coefficient λ_i can be reduced in two distinct ways [20, 27]: (1) by increasing

the homotypic cadherin bonds on cell-cell junctions or (2) by diminishing cortical contractile tension. Both scenarios would lead to a longer perimeter for the cell[20, 27].

The first two terms in Eq.1 can be combined into the well-known form[20, 24]: $K_P(P_i - P_0^i)^2$ where $P_0^i = -\lambda_i/(2K_P)$, an effective preferred perimeter[28] of the i-th cell. The third term in Eq 1 is associated with the energy cost for changing the cross-sectional area of the i-th cell from its rest area A_0^i with an elastic constant K_A. This energy arises due to cytoskeletal elasticity and cell deformations that change cell area away from A_0^i [21]. Together the perimeter and area energy terms give rise to the vertex model energy function[20, 21, 28] for N cells:

$$E = \sum_{i=1}^{N} [K_P(P_i - P_0)^2 + K_A(A_i - A_0)^2] \tag{1a}$$

For simplicity, here we treat all the cells as being mechanically identical. We can then rewrite Eq 1a in a dimensionless form as follows,

$$\tilde{E} = \sum_{i=1}^{N} \left[(p_i - p_0)^2 + \tilde{K}_A(a_i - 1)^2\right] \tag{1b}$$

wherein $K_P A_0$ is taken as the natural energy scale and $\sqrt{A_0}$ is taken as the natural length scale. The rescaled tissue energy, cell perimeter and cell area now become: $\tilde{E} = E/(K_P A_0)$, $p_i = P_i/\sqrt{A_0}$, $a_i = A_i/A_0$, respectively, and $\tilde{K}_A = K_A A_0/K_P$. The rescaled preferred cell perimeter becomes $p_0 = P_0/\sqrt{A_0}$ which is also called the target cell shape index[29]. When there is mismatch between the actual length of cell boundary (p_i) and this preferred value (p_0), tensions arise on cell edges. If the rescaled perimeters of two cells i and j sharing an edge are p_i and p_j, respectively, then the tension on the shared edge is given by:

$$T_{ij} = (p_i - p_0) + (p_j - p_0). \tag{2}$$

The Dynamic Vertex Model (DVM): To describe cellular migration, the rate of change of position of the i-th vertex, $\vec{r}_i$, at time t is represented by the overdamped equation of motion,

$$\Gamma \frac{d\vec{r}_i}{dt} = \vec{F}_i^{int} + v_0 \sum_{j \leftrightarrow i} w_j \hat{n}_j \tag{3}$$

where, Γ is the frictional damping. $\vec{F}_i^{\text{int}}$ describes those forces that arise due to cell-cell interactions and set by a spatial gradient in tissue mechanical energy: $-\partial E/\partial \vec{r}_i$. The last term represents an active motility force $\vec{f}_i$ on vertex i which has a magnitude v_0. Its direction is set by a weighted average of the polarization vectors of cells adjacent to vertex i, unlike a flat sum over the associated cell polarizations as described before[9, 30, 31]. The weight factor w_j for an adjacent cell j (polarization $\hat{n}_j$) is given by: $w_j = l_j/(2Z\Sigma_{j\leftrightarrow i}\, l_j)$ where l_j is the sum of the lengths of the two edges shared by cell j and vertex i, Z is the connectivity of vertex i and the division by 2 is done to avoid double counting. This ensures that the active force on any vertex gets the largest contribution from the neighboring cell that contains the longest edges connected to the vertex. We choose the cell polarization vectors in spirit of recent models of self-propelled particles[32-37]: $\hat{n}_j = (\cos\alpha_j, \sin\alpha_j)$ where the angle of polarization α_j follows over-damped dynamics according to:

$$\frac{d\alpha_j}{dt} = \eta_j, \langle \eta_j(t)\eta_k(t') \rangle = 2D_R \delta_{jk} \delta(t - t'). \tag{4}$$

This description models the front-back polarity that drives motility in migrating cells[29, 38, 39]. Each polarization vector is subjected to a Gaussian random noise η_j with zero mean and variance $2D_R$. Here D_R has units of inverse time and the we can define a cell-level persistence time $\tau_p = 1/D_R$ with units of time in the simulation and represents the persistence time for the polarization vector. For example, larger τ_p corresponds to slower remodeling for the front-back polarity of a cell and it would take approximately τ_p for polarization vector to reorient completely. Since v_0 is the self-propulsion force, then for a single cell (in the absence of cell-cell interactions), we can also define a persistence length

$$l_0 = \frac{v_0\, \tau_p}{\Gamma} \tag{5}$$

which serves to describe the distance (in units of a single cell's diameter) an individual unhindered cell will move before its direction changes. One important note is that in a confluent cell layer, cell-cell interactions will cause the actual observed persistence length to be much shorter than l_0.

Curving cell-cell boundaries: In this expanded version of the DVM, we now allow each edge to curve, as, as occurs during pEMT, by introducing a mid-edge vertex which provides an additional degree of freedom on each edge. Like the other vertices, each mid-edge vertex is subjected to active forces derived from the cell motility forces on the neighboring vertices. For the mid-edge vertex k on a given edge, the active force $\vec{f}_k$ is calculated using the active forces on the head and tail vertices of the edge using the following:

$$\vec{f}_k = \frac{1}{2}\left[\left(1 - \frac{l_1}{l_k}\right)\vec{f}_{k,1} + \left(1 - \frac{l_2}{l_k}\right)\vec{f}_{k,2}\right] \tag{6}$$

where $\vec{r}_k$ is the position vector of the mid-edge vertex, $\vec{f}_{k,1}$, $\vec{r}_{k,1}$ and $\vec{f}_{k,2}$, $\vec{r}_{k,2}$ are the active forces and position vectors of the head and tail vertices, respectively (Fig. S1). Also, $l_1 = |\vec{r}_k - \vec{r}_{k,1}|$, $l_2 = |\vec{r}_k - \vec{r}_{k,2}|$ represent the Euclidian distances of the head and tail vertices from the mid-edge vertex respectively, and $l_k = l_1 + l_2$, essentially the end-to-end length of the edge.

We estimate the contour length of an edge from an arc that represents the edge. The parametric equation for any point on the arc representing an edge is given by:

$$\vec{R}(s) = \vec{r}_{k,1} s\,(s-1)/2 + \vec{r}_k(1-s)(1+s) + \vec{r}_{k,2} s\,(s+1)/2 \tag{7}$$

where $-1 \le s \le 1$. This is implemented in the surface evolver program[40]. Because we keep the mean cell area fixed and cell-to-cell variations of area are small, pressure differences across cell edges are negligible. Therefore, when p_0 is small, the tissue is deep in the solid state, and the edges are under high tension and hence straight. However, for large p_0 the edge tension diminishes, and edges curve to accommodate the large perimeter.

Simulation details: We initialized each simulation using independent Voronoi tessellation of randomly placed points posing as cell centers. This gives rise to a confluent polygonal tiling that is random. Then we minimized tissue energy (Eq 1b) to find a state with energy close to the ground state energy at zero motility ($v_0 = 0$) using the conjugate gradient protocol with respect to the vertex locations. Next, we allow finite motility i.e. $v_0 > 0$ and performed the dynamical simulations. The vertex locations are updated by the Euler method using Eq. 3 with a timestep of $\Delta t = 10^{-2}\tau$ where $\tau = \Gamma/K_P$, the unit of time in the DVM. All lengths in DVM was measured in unit of $\sqrt{A_0}$ where we use $A_0 = \bar{A}$, the mean area of the cells which is maintained at unity throughout any simulation. The rotational noise on the direction of cell polarizations is given by Eq. 4.

We determined the effects of independently varying the magnitude of cellular motility force, v_0, the preferred cell perimeter, p_0 and the persistence length of an isolated cell l_0. When keeping all other parameters fixed, we found that increasing only p_0 recapitulated observations made during pEMT, while increasing only v_0 recapitulated observations made during UJT. For these two cases we used a fixed and reasonably high rotational noise

strength $D_R = 0.5$, so that the single cell persistence length l_0 remains small (a few cell diameters or less). Furthermore, when we increased only l_0, by reducing the strength of rotational noise D_R at fixed $p_0 = 4$ and $v_0 = 1.2$, we could capture the increase in dynamic cell pack sizes observed during the progress of UJT. Note that each of our simulations depend on a single set of v_0, p_0 and l_0 values which do not change during the simulation. Moreover, for each parameter set we ran 10-20 different simulations from independent initial configurations. Thus, all the error bars associated with data from the simulations (Fig. 4 and Extended Data Figure 6) are SOM, generated from these different independent trajectories.

From the independent simulations corresponding to each set of v_0, p_0 and l_0 values, we calculated the following average quantities across the cells in the simulated tissue: edge tension (Eq. 2), edge tortuosity (defined in main text *Methods*), aspect ratio, cell shape index[9, 29] (defined for cell i as $q_i = P_i/\sqrt{A_i} = p_i/\sqrt{a_i}$), velocities, effective diffusivities and the dynamic cell pack sizes from the cell velocities. The aspect ratio of any cell in the tissue was calculated from the eigenvalues of the shape tensor[25], generated using the positions of the vertices of the cell.

In a confluent epithelial sheet the basic mode of migration is T1 transitions[41] in which cells swap positions with their local neigbors[42, 43]. In the DVM we allow T1 rearrangements by implementing an embargo timer on each cell given by parameter τ_{T1}. This sets a lower limit on the time between successive T1s involving a cell. We chose appropriate values for τ_{T1} in different simulation scenarios. For simulations with $v_0 > 0.05$ (associated with UJT) we use $\tau_{T1} = \tau$, while we use a much larger value $\tau_{T1} = 10^3\tau$ for the simulations at $v_0 = 0.05$ (associated with pEMT where T1 processes were rarely observed in the experiments). The threshold length for T1 edge swap was fixed at $l_c = 0.1$ in simulation length unit. We used periodic boundary conditions in both x and y directions on our simulation box containing $N = 400$ cells. All our simulations were implemented using the Surface-Evolver program[40].

<u>Analysis of dynamic pack sizes from DVM</u>: The dynamic pack sizes from the DVM (Fig. 4e, f and h) were computed using the same algorithm employed for the experimental cell velocity fields (Fig. 3), but with a different angle cut-off of 30 degrees for defining nearest neighbors of a given cell and considering only the cells with speeds larger than a fixed cutoff of 0.02 in simulation unit of speed ($K_p\sqrt{A_0}/\Gamma$). The cell speeds were calculated from the simulated trajectories using displacements over a time interval. This time interval is chosen suitably to reduce the contribution from very short-lived transient packs, predominantly observed when l_0 is comparable to one cell diameter or less. Due to the periodic boundary conditions used in DVM, we extracted the linear sizes of the dynamic packs along the x and y axes by mapping the individual cartesian coordinates of all the cell vertices on two separate one-dimensional grids along x and y axes, each with spacing $ds \approx 0.4$ cell diameter (illustrated in Fig. S2). After binning the respective coordinates in these two grids, if the histogram bin-counts along x and y axes are h_x and h_y respectively (Fig. S2), then the linear size of the pack is given by: $l_p = \max(l_x, l_y)$ where $l_x = \sum_x sgn(h_x)\, ds$ and $l_y = \sum_y sgn(h_y)\, ds$. For each configuration of dynamic packs the mean pack size per cell was calculated just like in the experimental data: if the j-th cell is in a dynamic pack with length l_j, and there are N cells, the mean linear dynamic pack size per cell would be $\langle l_p \rangle = (1/N)\sum_{j=1}^{N} l_j$. We also recorded the maximum pack sizes, the variation of which as a function of l_0 is shown in Fig. 4f, while the mean pack sizes are shown in Fig. 4h.

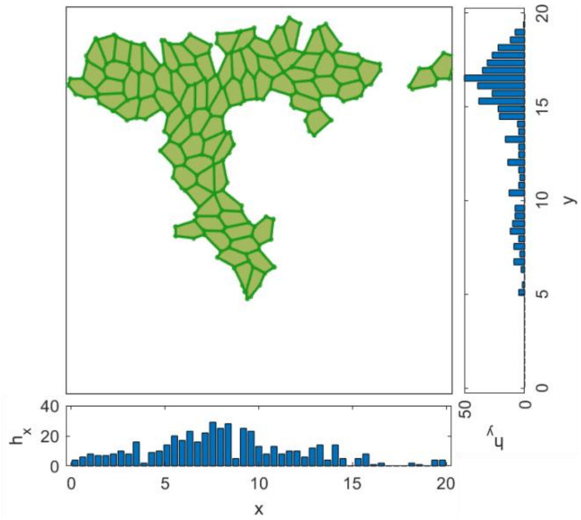

Figure S2. Illustration of the method used to determine the linear size of a dynamic pack from DVM.

References

1. Park, J.A., Fredberg, J.J. & Drazen, J.M. Putting the Squeeze on Airway Epithelia. *Physiology (Bethesda)* **30**, 293-303 (2015).
2. Tschumperlin, D.J. *et al.* Mechanotransduction through growth-factor shedding into the extracellular space. *Nature* **429**, 83-86 (2004).
3. Grainge, C.L. *et al.* Effect of bronchoconstriction on airway remodeling in asthma. *N Engl J Med* **364**, 2006-2015 (2011).
4. Wiggs, B.R., Hrousis, C.A., Drazen, J.M. & Kamm, R.D. On the mechanism of mucosal folding in normal and asthmatic airways. *J Appl Physiol (1985)* **83**, 1814-1821 (1997).
5. Swartz, M.A., Tschumperlin, D.J., Kamm, R.D. & Drazen, J.M. Mechanical stress is communicated between different cell types to elicit matrix remodeling. *Proc Natl Acad Sci U S A* **98**, 6180-6185 (2001).
6. Tschumperlin, D.J. & Drazen, J.M. Chronic effects of mechanical force on airways. *Annu Rev Physiol* **68**, 563-583 (2006).
7. Tschumperlin, D.J. & Drazen, J.M. Mechanical stimuli to airway remodeling. *Am J Respir Crit Care Med* **164**, S90-94 (2001).
8. Park, J.A. & Tschumperlin, D.J. Chronic intermittent mechanical stress increases MUC5AC protein expression. *Am J Respir Cell Mol Biol* **41**, 459-466 (2009).
9. Park, J.A. *et al.* Unjamming and cell shape in the asthmatic airway epithelium. *Nat Mater* **14**, 1040-1048 (2015).
10. Park, J.A., Drazen, J.M. & Tschumperlin, D.J. The chitinase-like protein YKL-40 is secreted by airway epithelial cells at base line and in response to compressive mechanical stress. *J Biol Chem* **285**, 29817-29825 (2010).
11. Park, J.A. *et al.* Tissue factor-bearing exosome secretion from human mechanically stimulated bronchial epithelial cells in vitro and in vivo. *J Allergy Clin Immunol* **130**, 1375-1383 (2012).
12. Tschumperlin, D.J., Shively, J.D., Kikuchi, T. & Drazen, J.M. Mechanical stress triggers selective release of fibrotic mediators from bronchial epithelium. *Am J Respir Cell Mol Biol* **28**, 142-149 (2003).
13. Kilic, A. *et al.* Mechanical forces induce an asthma gene signature in healthy airway epithelial cells. *Sci Rep* **10**, 966 (2020).

14. Kim, S.H. *et al.* Increased extracellular maspin levels after mechanical compression in vitro or allergen challenge in vivo. *J Allergy Clin Immunol* **144**, 1116-1118 e1114 (2019).
15. Lan, B. *et al.* Airway epithelial compression promotes airway smooth muscle proliferation and contraction. *Am J Physiol Lung Cell Mol Physiol* (2018).
16. Mitchel, J.A. *et al.* IL-13 Augments Compressive Stress-Induced Tissue Factor Expression in Human Airway Epithelial Cells. *Am J Respir Cell Mol Biol* **54**, 524-531 (2016).
17. Nagai, T. & Honda, H. A dynamic cell model for the formation of epithelial tissues. *Philosophical Magazine B-Physics of Condensed Matter Statistical Mechanics Electronic Optical and Magnetic Properties* **81**, 699-719 (2001).
18. Fletcher, A.G., Osterfield, M., Baker, R.E. & Shvartsman, S.Y. Vertex models of epithelial morphogenesis. *Biophys J* **106**, 2291-2304 (2014).
19. Honda, H. Description of cellular patterns by Dirichlet domains: the two-dimensional case. *J Theor Biol* **72**, 523-543 (1978).
20. Farhadifar, R., Roper, J.C., Aigouy, B., Eaton, S. & Julicher, F. The influence of cell mechanics, cell-cell interactions, and proliferation on epithelial packing. *Curr Biol* **17**, 2095-2104 (2007).
21. Hufnagel, L., Teleman, A.A., Rouault, H., Cohen, S.M. & Shraiman, B.I. On the mechanism of wing size determination in fly development. *Proc Natl Acad Sci U S A* **104**, 3835-3840 (2007).
22. Brodland, G.W. *et al.* Video force microscopy reveals the mechanics of ventral furrow invagination in Drosophila. *Proc Natl Acad Sci U S A* **107**, 22111-22116 (2010).
23. Chiou, K.K., Hufnagel, L. & Shraiman, B.I. Mechanical stress inference for two dimensional cell arrays. *PLoS Comput Biol* **8**, e1002512 (2012).
24. Staple, D.B. *et al.* Mechanics and remodelling of cell packings in epithelia. *Eur Phys J E Soft Matter* **33**, 117-127 (2010).
25. Atia, L. *et al.* Geometric constraints during epithelial jamming. *Nature Physics* **14**, 613-620 (2018).
26. Malinverno, C. *et al.* Endocytic reawakening of motility in jammed epithelia. *Nat Mater* **16**, 587-596 (2017).
27. Manning, M.L., Foty, R.A., Steinberg, M.S. & Schoetz, E.M. Coaction of intercellular adhesion and cortical tension specifies tissue surface tension. *Proc Natl Acad Sci U S A* **107**, 12517-12522 (2010).
28. Bi, D.P., Lopez, J.H., Schwarz, J.M. & Manning, M.L. A density-independent rigidity transition in biological tissues. *Nature Physics* **11**, 1074-+ (2015).
29. Bi, D., Yang, X., Marchetti, M.C. & Manning, M.L. Motility-driven glass and jamming transitions in biological tissues. *Phys Rev X* **6** (2016).
30. Czajkowski, M., Sussman, D.M., Marchetti, M.C. & Manning, M.L. Glassy Dynamics in Models of Confluent Tissue with Mitosis and Apoptosis. *eprint arXiv:1905.01603*, arXiv:1905.01603 (2019).
31. Hirashima, T. & Adachi, T. Anisotropic Cellular Mechanoresponse for Radial Size Maintenance of Developing Epithelial Tubes. *bioRxiv*, 172916 (2017).
32. Fily, Y., Henkes, S. & Marchetti, M.C. Freezing and phase separation of self-propelled disks. *Soft Matter* **10**, 2132-2140 (2014).
33. Fily, Y. & Marchetti, M.C. Athermal Phase Separation of Self-Propelled Particles with No Alignment. *Physical Review Letters* **108**, 235702 (2012).
34. Cates, M.E. & Tailleur, J. Motility-Induced Phase Separation. *Annual Review of Condensed Matter Physics, Vol 6* **6**, 219-244 (2015).
35. Chate, H., Ginelli, F., Gregoire, G. & Raynaud, F. Collective motion of self-propelled particles interacting without cohesion. *Phys Rev E Stat Nonlin Soft Matter Phys* **77**, 046113 (2008).
36. Garcia, S. *et al.* Physics of active jamming during collective cellular motion in a monolayer. *Proc Natl Acad Sci U S A* **112**, 15314-15319 (2015).
37. Bechinger, C. *et al.* Active Particles in Complex and Crowded Environments. *Reviews of Modern Physics* **88** (2016).
38. Mayor, R. & Etienne-Manneville, S. The front and rear of collective cell migration. *Nat Rev Mol Cell Biol* **17**, 97-109 (2016).

39. Szabo, A. *et al.* Collective cell motion in endothelial monolayers. *Physical biology* **7**, 046007 (2010).
40. Brakke, K.A. The surface evolver. *Experiment. Math.* **1**, 141-165 (1992).
41. Weaire, D. & Rivier, N. Soap, Cells and Statistics - Random Patterns in 2 Dimensions. *Contemp Phys* **25**, 59-99 (1984).
42. Bertet, C., Sulak, L. & Lecuit, T. Myosin-dependent junction remodelling controls planar cell intercalation and axis elongation. *Nature* **429**, 667-671 (2004).
43. Zallen, J.A. & Wieschaus, E. Patterned gene expression directs bipolar planar polarity in Drosophila. *Dev Cell* **6**, 343-355 (2004).
