# Supplementary material for: In primary airway epithelial cells, the unjamming transition is distinct from the epithelial-to-mesenchymal transition
Source: Nat Commun. 2020 Oct 7;11:5053. doi: 10.1038/s41467-020-18841-7 (PMC7542457; doi:10.1038/s41467-020-18841-7)
Supplement: Supplementary file 3 — Reporting Summary [file 41467_2020_18841_MOESM3_ESM.pdf]

nature research

Corresponding author(s): Jin-Ah Park

Last updated by author(s): Aug 6, 2020

Reporting Summary

Nature Research wishes to improve the reproducibility of the work that we publish. This form provides structure for consistency and transparency in reporting. For further information on Nature Research policies, see Authors & Referees and the Editorial Policy Checklist .

Statistics

For all statistical analyses, confirm that the following items are present in the figure legend, table legend, main text, or Methods section.

n/a	Confirmed	
☐	☒	The exact sample size (*n*) for each experimental group/condition, given as a discrete number and unit of measurement
☐	☒	A statement on whether measurements were taken from distinct samples or whether the same sample was measured repeatedly
☐	☒	The statistical test(s) used AND whether they are one- or two-sided<br>*Only common tests should be described solely by name; describe more complex techniques in the Methods section.*
☒	☐	A description of all covariates tested
☐	☒	A description of any assumptions or corrections, such as tests of normality and adjustment for multiple comparisons
☐	☒	A full description of the statistical parameters including central tendency (e.g. means) or other basic estimates (e.g. regression coefficient) AND variation (e.g. standard deviation) or associated estimates of uncertainty (e.g. confidence intervals)
☐	☒	For null hypothesis testing, the test statistic (e.g. *F*, *t*, *r*) with confidence intervals, effect sizes, degrees of freedom and *P* value noted<br>*Give P values as exact values whenever suitable.*
☒	☐	For Bayesian analysis, information on the choice of priors and Markov chain Monte Carlo settings
☒	☐	For hierarchical and complex designs, identification of the appropriate level for tests and full reporting of outcomes
☒	☐	Estimates of effect sizes (e.g. Cohen's *d*, Pearson's *r*), indicating how they were calculated

Our web collection on statistics for biologists contains articles on many of the points above.

Software and code

Policy information about availability of computer code

Data collection	Phase and fluorescent images were collected using ZEN blue (v. 2.0) software. Cell shape data was collected using SeedWater Segmenter software (v0.5.7.1). Western blot and mRNA data were collected as described in the methods section.
Data analysis	Custom code written in Matlab (R2019a) was used to determine dynamic and structural data from phase and fluorescent images. Cellular trajectories were generated using the optical flow (farneback) functions built into Matlab (R2019a). Images were processed in Zen blue (v. 2.0.) and ImageJ (v 1.52n). All software is available upon request.

For manuscripts utilizing custom algorithms or software that are central to the research but not yet described in published literature, software must be made available to editors/reviewers. We strongly encourage code deposition in a community repository (e.g. GitHub). See the Nature Research guidelines for submitting code & software for further information.

Data

Policy information about availability of data

All manuscripts must include a data availability statement. This statement should provide the following information, where applicable:

- Accession codes, unique identifiers, or web links for publicly available datasets
- A list of figures that have associated raw data
- A description of any restrictions on data availability

Data that comprise the graphs within this manuscript are included in the Source Data file. Any findings or raw data of this study are available from the corresponding author upon request.

Field-specific reporting

Please select the one below that is the best fit for your research. If you are not sure, read the appropriate sections before making your selection.

- [x] Life sciences
- [ ] Behavioural & social sciences
- [ ] Ecological, evolutionary & environmental sciences

For a reference copy of the document with all sections, see nature.com/documents/nr-reporting-summary-flat.pdf

Life sciences study design

All studies must disclose on these points even when the disclosure is negative.

Sample size	Sample size calculations were not performed, but we chose to use 3-4 independent donors based on our previous studies (Park et al, Nature Materials 2015). All studies were performed with at least n=3 independent experimental replications. These final studies were carried out after extensive preliminary data was collected.
Data exclusions	Dynamic analysis for certain data points was excluded in the case when there was extreme drift in the sample on the microscope which could not be corrected by standard drift correction methods. These were excluded before the analysis was performed, based on the observation of the extreme drift. This occurred rarely and randomly.
Replication	We use primary airway epithelial cells from non-diseased patients. We replicated our studies in cells derived from at least 3-4 donors, and the data presented in the manuscript are taken in toto from all of these experiments.
Randomization	In all experiments, internal controls were present for each donor, and we present results across donors. Wells were assigned to control, compression, or TGF-beta-1 treatment groups randomly. To control for our mechanical compression condition, we exposed the control cells to a sham pressure setup. To control for our exposure to TGF-beta-1, we exposed the control cells to vehicle.
Blinding	Where possible, researchers were blinded. In all cases, the same analysis process was applied to all samples, regardless of blinding. Researchers were blinded when isolating mRNA and when measuring the dynamical (speed, diffusivity, dynamic pack size) and structural (aspect ratio, orientation pack size) properties of the samples.

Reporting for specific materials, systems and methods

We require information from authors about some types of materials, experimental systems and methods used in many studies. Here, indicate whether each material, system or method listed is relevant to your study. If you are not sure if a list item applies to your research, read the appropriate section before selecting a response.

Materials & experimental systems

n/a	Involved in the study	
[ ]	[x]	Antibodies
[ ]	[x]	Eukaryotic cell lines
[x]	[ ]	Palaeontology
[x]	[ ]	Animals and other organisms
[ ]	[x]	Human research participants
[x]	[ ]	Clinical data

Methods

n/a	Involved in the study	
[x]	[ ]	ChIP-seq
[x]	[ ]	Flow cytometry
[x]	[ ]	MRI-based neuroimaging

Antibodies

Antibodies used	The following antibodies and dilutions were used for Western blot analysis: E-cadherin (1:10,000, Cell Signaling Technology #3195), N-cadherin (1:1000, Cell Signaling Technology, #13116), Snail1 (1:1000, Cell Signaling Technology, #3879), vimentin (1:1000, Cell Signaling Technology, #5741), GAPDH (1:5000, Cell Signaling Technology, #5174), EDA-fibronectin (1:1000, Sigma-Millipore #MAB1940). The following antibodies and dilutions were used for immunocytochemistry: E-cadherin (1:200, Cell Signaling Technology #3195), ZO-1 (1:100, ThermoFisher, clone ZO1-1A12, #33-9100), vimentin (1:100, Cell Signaling Technology #5741), cellular fibronectin (1:200, Sigma-Millipore #MAB1940)
Validation	All antibodies were used according to validation on the manufacturer's website.<br>Western blotting:<br>The antibody against E-cadherin was purchased from Cell Signaling Technology (CST), which validated the Ab for human E-cadherin by WB. Using this antibody at the dilution ratio recommended by CST, and at lower dilutions, we detected E-cadherin protein at the expected MW (135kDa) using human cell lysates.<br>The antibody against N-cadherin was purchased from Cell Signaling Technology (CST), which validated the Ab for human N-cadherin by WB. Using this antibody at the dilution ratio recommended by CST (1:1000), we detected N-cadherin protein at the expected MW (140kDa) using human cell lysates.<br>The antibody against vimentin was purchased from CST, which validated the Ab for human vimentin by WB. Using this antibody

at the dilution ratio recommended by CST (1:1000), we detected vimentin protein at the expected MW (60kDa) using human cell lysates.
The antibody against Snail1 was purchased from CST, which validated the Ab for human Snail1 by WB. Using this antibody at the dilution ratio recommended by CST (1:1000), we detected Snail1 protein at the expected MW (29kDa) using human cell lysates.
The antibody against GAPDH was purchased from CST, which validated the Ab for human GAPDH by WB. Using this antibody at lower than the dilution ratio recommended by CST (we used 1:5000), we detected GAPDH protein at the expected MW (34kDa) using human cell lysates.
The antibody against EDA-fibronectin (FN) was purchased from Sigma-Millipore, which validated the Ab for human FN by WB. Using this antibody at the ratio recommended by CST (1:1000), we detected FN protein at the expected MW (250kDa) using human cell lysates.

Immunofluroescence:
The antibody against E-cadherin was purchased from CST, which validated for human E-cadherin by IF. We used fixed human cells to validate the E-cadherin antibody, ensuring that positive signal appeared in the expected location (junctional protein). We used IgG as a negative control.

The antibody against ZO1 was purchased from Thermofisher, which validated for human ZO1 by IF. We used fixed human cells to validate the ZO1 antibody, ensuring that positive signal appeared in the expected location (junctional protein). We used IgG as a negative control.

The antibody against vimentin was purchased from Thermofisher, which validated for human vimentin by IF.
The antibody against FN was purchased from Thermofisher, which validated for human FN by IF.
For both vimentin and FN, we used fixed human fibroblasts to validate the antibody. This cell type is mesenchymal and known to express high levels of vimentin and FN. We used IgG as a negative control.

Eukaryotic cell lines

Policy information about cell lines

Cell line source(s)	We used primary human epithelial cells. See the "human research participants" section, below, for additional details.
Authentication	These primary cells were not authenticated by any external source.
Mycoplasma contamination	These primary cells were not tested for mycoplasma.
Commonly misidentified lines (See ICLAC register)	N/A

Human research participants

Policy information about studies involving human research participants

Population characteristics	We used primary human epithelial cells. Primary HBECs were isolated at Passage 0 at the Marsico Lung Institute/Cystic Fibrosis Research Center at the University of North Carolina, Chapel Hill. Human lungs unsuitable for transplanataion were obtained under protocol #03-1396 approved by the University of North Carolina at Chapel Hill Biomedical Institutional Review Board. Informed consent was obtained from authorized representatives of all organ donors. Lungs were from non-smokers with no history of chronic lung disease. Demographic information is available for all donors used in our study upon request. Cells were expanded to Passage 2 in our lab, and used for all experiments at Passage 2, as described in the manuscript.
Recruitment	Recruitment was performed at UNC Chapel Hill as stated above.
Ethics oversight	Human lungs unsuitable for transplanataion were obtained under protocol #03-1396 approved by the University of North Carolina at Chapel Hill Biomedical Institutional Review Board.

Note that full information on the approval of the study protocol must also be provided in the manuscript.
